# Expanding Housing and Support Eligibility by Veteran Discharge Status

**DOI:** 10.1001/jamanetworkopen.2025.25202

**Published:** 2025-08-05

**Authors:** Thomas F. Nubong, Jack Tsai, Christopher Halladay, James L. Rudolph, Eric Jutkowitz

**Affiliations:** 1 Center of Innovation for Long-Term Services and Supports, Providence Veterans Affairs Medical Center, Providence, Rhode Island; 2 Brown University School of Public Health, Providence, Rhode Island; 3 VA National Center on Homelessness Among Veterans, Washington, DC

## Abstract

This quality improvement study examines enrollment outcomes among veterans after policy changes to the US Department of Housing and Urban Development Veterans Affairs Supportive Housing program.

## Introduction

The US Department of Housing and Urban Development–Veterans Affairs Supportive Housing (HUD-VASH) program provides housing support and case management services to veterans experiencing homelessness.^1,2^ Before 2021, with exceptions, veterans with an other-than-honorable (OTH) discharge (ie, military discharge due to misconduct), were ineligible for most US Department of Veterans Affairs (VA) services, including HUD-VASH. On January 1, 2021, the US Congress passed the National Defense Authorization Act for Fiscal Year 2021 (P.L. 116-283), allowing the VA to provide HUD-VASH and other homeless services to OTH veterans.^3^ We investigated HUD-VASH outcomes before and after the policy, hypothesizing an increase in enrollments of OTH veterans and no adverse outcomes on services for honorable and/or general discharge veterans.

## Methods

This quality improvement study is reported in accordance with the SQUIRE 2.0 reporting guidelines and was determined by the VA Homeless Programs Office to not constitute human participants research; thus, informed consent was not needed, in accordance with 45 CFR §46. We linked data from the Homeless Operations and Management Evaluation System database with the VA Corporate Data Warehouse to identify veterans enrolled in HUD-VASH from June 1, 2019, to September 30, 2021, and their discharge status. Using an interrupted time series design and segmented linear regression, we compared HUD-VASH enrollments, emergency department (ED) visits, hospitalizations, and primary care visits before and after P.L. 116-283 at the program-month level (June 2019 to December 2020 vs January 2021 to September 2021).^4,5,6^ A total of 2 526 (1.9%) of the initial cohort was excluded due to punitive and/or missing discharges. All statistical analyses were conducted using R version 4.4.1. Statistical signifcance was assessed at p < .05. We did not perform covariate adjustment because veteran characteristics were similar before and after P.L. 116-283.

## Results

**Figure 1.**
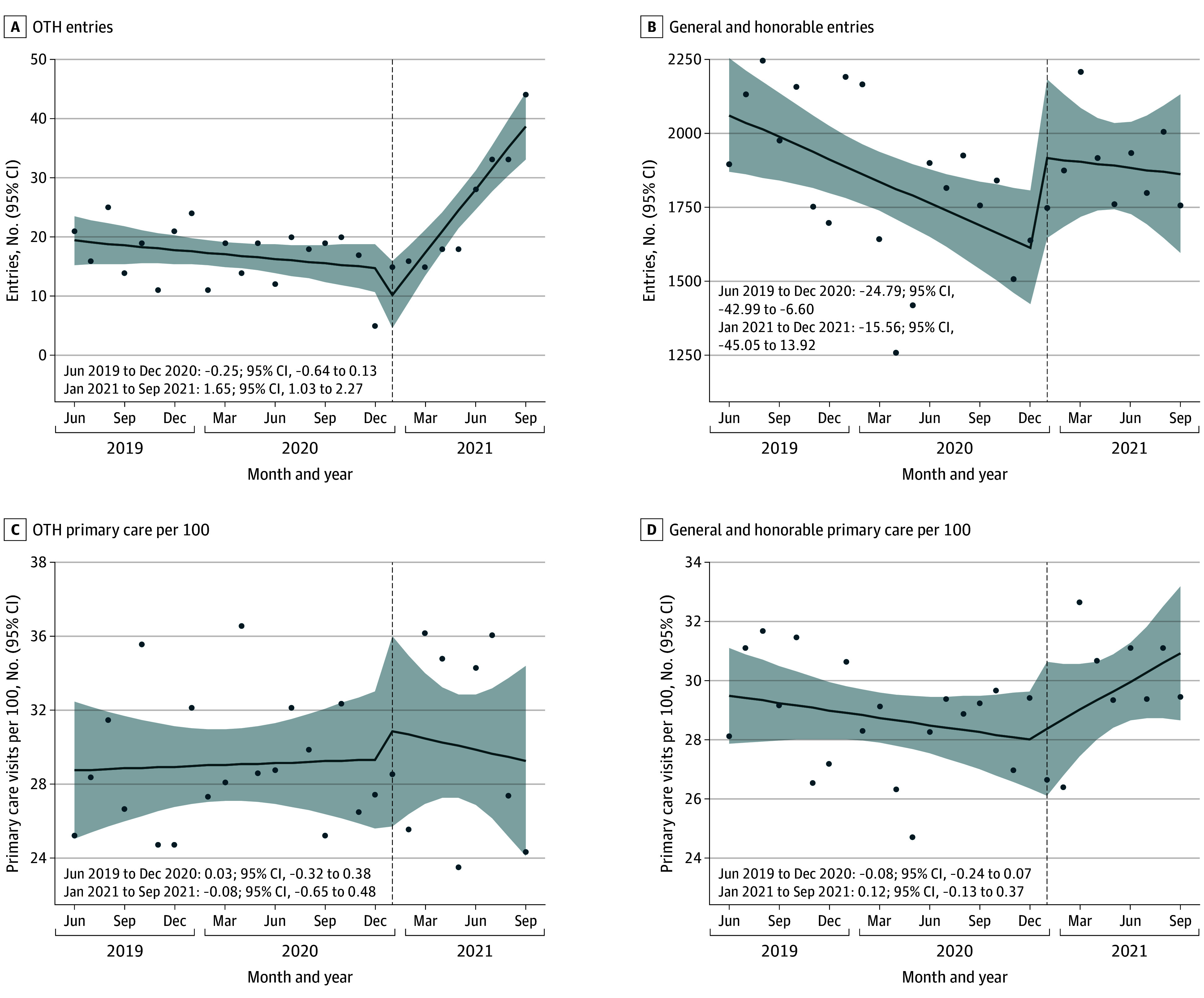
Trends in US Department of Housing and Urban Affairs Veterans Affairs Supportive Housing Program Entries by Discharge Status Shaded areas denote 95% CIs. OTH indicates other than honorable. Dots represent monthly observed values for entries and exits, or monthly mean values for all other measures.

**Figure 2.**
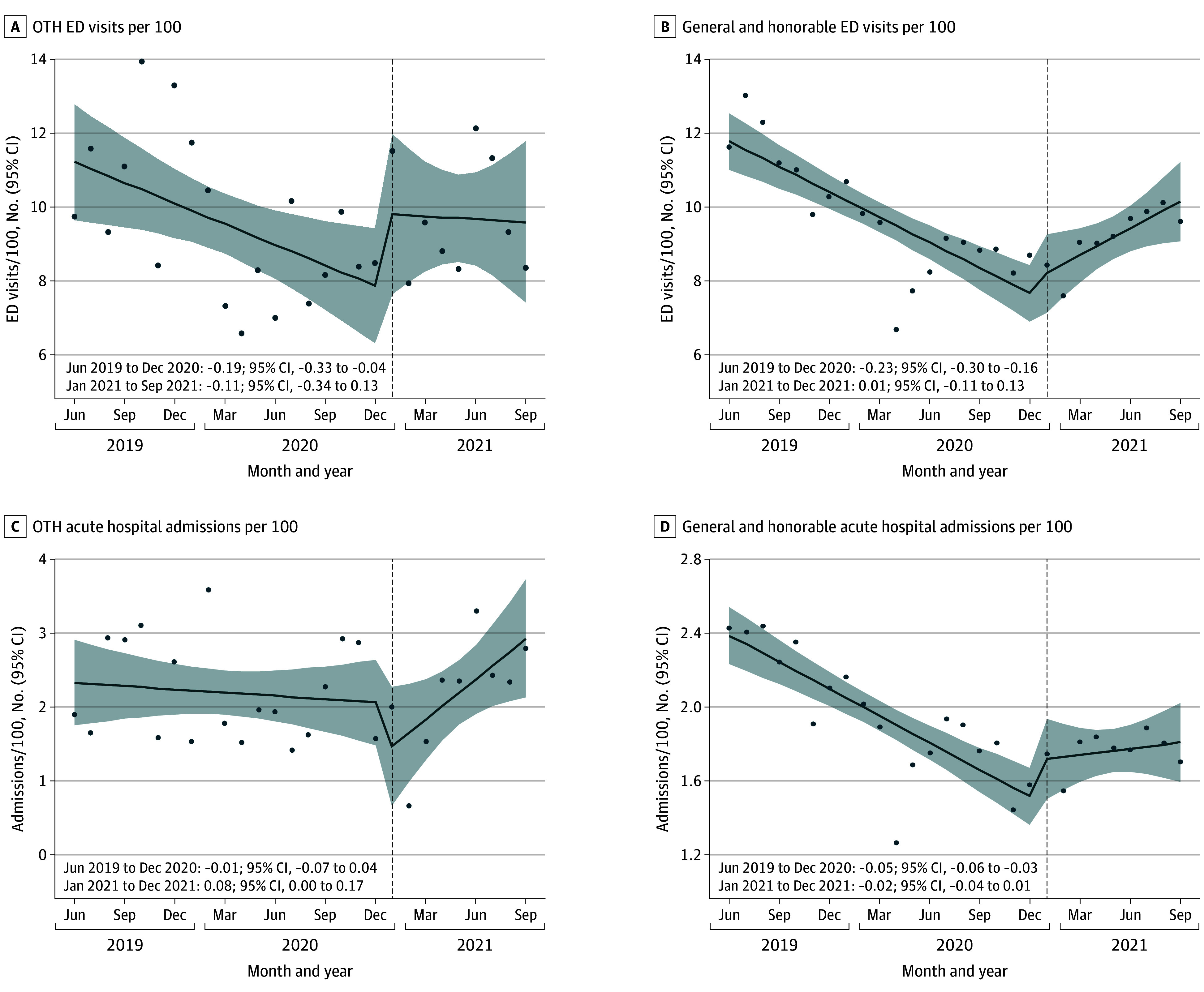
Utilization of Health Care Services Among Veterans by Discharge Status Shaded areas denote 95% CIs. ED indicates emergency department; and OTH, other than honorable. Dots represent monthly observed values for entries and exits, or monthly mean values for all other measures.

A total of 129 873 veterans were enrolled in HUD-VASH during the study period, with 127 876 (98.5%) having an honorable and/or general discharge and 1997 (1.5%) having an OTH discharge. Veterans with both groups were predominantly male. Overall, a mean (SD) of 87% (34) were male (approximately 112 980 individuals); 13% (34) were female (approximately 16 884). Among those with OTH discharges, a mean (SD) of 94% (23) were male (approximately 1877 individuals) and 6% (23) were female (approximately 120 individuals). The mean age was 53.71 (SD, 13.55) years for the overall cohort and 53.29 (SD, 11.90) years among those with OTH discharges. In Figure 1A, there was a significant increase in monthly HUD-VASH enrollments after compared with before the policy for OTH veterans (difference in slopes, 1.90; 95% CI, 1.28 to 2.52). In Figure 1B there was an increase in HUD-VASH enrollments after vs before the policy change for honorable and/or general veterans, but the difference was not statistically significant (difference in slopes, 9.23; 95% CI, −20.35 to 38.79). In Figure 1C and 1D, there was no significant difference in primary care visits after compared with before the policy change for both OTH (change in slope, −0.12; 95% CI, −0.65 to 0.42) and honorable and/or general veterans (change in slope, 0.20; 95% CI, −0.13 to 0.53). In Figure 2A and 2B, ED visits declined before the policy change for both groups. There was small but not statistically significant increase in ED visits after compared with before the policy change for OTH veterans (change in slope, 0.08; 95% CI, −0.12 to 0.28) and a significant increase for honorable and/or general veterans (change in slope, 0.24; 95% CI, 0.12 to 0.35). In Figure 2C and 2D, hospitalizations significantly increased after compared with before the policy for both OTH (change in slope, 0.098; 95% CI, 0.009 to 0.170) and honorable and/or general veterans (change in slope, 0.078; 95% CI, 0.004 to 0.060).

## Discussion

This quality improvement study found that after P.L. 116-283, HUD-VASH enrollments for OTH veterans significantly increased without crowding out enrollments for honorable and/or general veterans. ED visits increased for honorable and/or general veterans, while hospitalizations increased for both groups. The increase in hospitalizations for OTH veterans and no change in primary care visits may suggest that these veterans continued to face difficulties accessing VA outpatient services beyond those newly extended under P.L. 116-283.

This study has limitations that should be mentioned. The study period overlaps with the COVID-19 pandemic, and staff training on the policy change varied across VA sites. We used a single-group interrupted time series design to assess within-group changes. While this approach captures temporal trends, the absence of a multigroup design limits formal comparisons between discharge groups.

In conclusion, expanding HUD-VASH eligibility increased access to housing and social support for OTH veterans without disrupting services for those with honorable discharges. Efforts should focus on improving access to connecting OTH veterans with clinical services outside of HUD-VASH.

## Notes

Supplement.
Data Sharing Statement
