# Supplementary material for: Expanding Housing and Support Eligibility by Veteran Discharge Status
Source: JAMA Netw Open. 2025 Aug 5;8(8):e2525202. doi: 10.1001/jamanetworkopen.2025.25202 (PMC12326279; doi:10.1001/jamanetworkopen.2025.25202)
Supplement: Supplement. — Data Sharing Statement [file jamanetwopen-e2525202-s001.pdf]

Data Sharing Statement

Nubong. Expanding Housing and Support Eligibility by Veteran Discharge Status. *JAMA Netw Open*. Published August 05, 2025. doi:10.1001/jamanetworkopen.2025.25202

Data
Data available: No
